# Evaluation of Triglyceride Glucose Index in Patients with Patent Foramen Ovale Who Experienced Cryptogenic Stroke

**DOI:** 10.3390/jcm13237271

**Published:** 2024-11-29

**Authors:** Burak Ayça, Cennet Yıldız, Yasin Yüksel, Fahrettin Katkat, Atakan Arpaç, Fatma Nihan Turhan Çağlar, Cansu Erkol

**Affiliations:** 1 Cardiology Department, Istanbul Training and Research Hospital, Istanbul 34098, Turkey; drburakayca@yahoo.com.tr (B.A.); fahrettin_katkat@hotmail.com (F.K.); 2 Cardiology Department, Bakırkoy Dr. Sadi Konuk Training and Research Hospital, Istanbul 34147, Turkey; atakan.arpac@gmail.com (A.A.); nhnturhan@gmail.com (F.N.T.Ç.); 3 Cardiology Department, Private Reyap Hospital, Istanbul 34515, Turkey; dryasinyuksel@gmail.com; 4 Department of Neurology, Istanbul Training and Education Hospital, Istanbul 34098, Turkey; cansuerkol@gmail.com

**Keywords:** patent foramen ovale, stroke, risk factors, triglyceride, glucose

## Abstract

**Background/Objectives**: The prevalence of patent foramen ovale (PFO) has been found to be increased in patients with cryptogenic stroke, suggesting an association between these two clinical settings. Insulin resistance is a risk factor for the occurrence of stroke. The triglyceride glucose (TyG) index is a biomarker that reflects the IR status of the body. Our aim was to evaluate the TyG index values in patients with PFO who experienced cryptogenic stroke. **Methods**: One hundred and twenty nine patients with PFO who experienced embolic stroke and one hundred and eight control subjects were enrolled. All patients in the study group experienced embolic stroke within 2 weeks of enrollment. The TyG index value of each patient was calculated. **Results**: Patients with stroke were significantly older, had higher levels of glucose, creatinine, triglyceride (TG), leukocyte, and TyG index and lower high-density lipoprotein–cholesterol values. The TyG index had the highest sensitivity for the prediction of stroke in comparison to TG and glucose values. Comparison of ROC curves showed that the TyG index had the highest AUC compared to that of TG and glucose. The TyG index value of 8.89 predicted stroke occurrence with a sensitivity and specificity of 63.2% and 72.3%, respectively. The results of multivariable regression analyses showed that the TyG index had a higher odds ratio than TG, which indicated that it had a better predictive value. **Conclusions**: Assessment of the TyG index in cryptogenic stroke patients with PFO might be helpful for the management of these patients.

## 1. Introduction

Despite improvements in the management of patients and improved risk factor control, stroke has remained the second leading cause of death and a major cause of disability worldwide [1]. Ischemic stroke accounts for approximately eighty percent of stroke cases and has risk factors, including hypertension, age, family history, diabetes mellitus, atrial fibrillation, and smoking history. In addition to these risk factors, other risk factors, such as insulin resistance (IR), have been linked with the occurrence of stroke [2]. According to previous studies, ischemic stroke and type 2 diabetes mellitus (DM) share common causative factors. It has been found that almost half of nondiabetic patients diagnosed with transient ischemic attack or ischemic stroke have IR [3]. Twenty percent of nondiabetic ischemic stroke patients had elevated HOMA-R values [4]. IR could cause ischemic stroke through platelet adhesion and aggregation, thrombus formation, and promoting the formation and progression of atherosclerotic plaques [5,6,7]. IR is associated with decreased nitric oxide and increased endothelin-1 production with resultant endothelial dysfunction [7]. Increased free fatty acid release may lead to higher expression of coagulative tissue factors [8]. The anti-inflammatory activity of high-density lipoprotein cholesterol (HDL-C) is impaired in the IR state, and levels of inflammatory factors such as C-reactive protein, interleukin, are elevated [9]. Studies have demonstrated that IR leads to dyslipidemia [10]. Excessive production of very-low-density lipoprotein and small dense low-density lipoprotein cholesterol (LDL-C) accelerate atherosclerotic process [11]. Mitogen-activated protein kinase pathway activated with resultant increase in cell adhesion aids in atherosclerosis [12]. The major cause of ischemic stroke is atherosclerosis, and it is most frequently caused by embolism. IR, by initiating and progression of atherosclerotic process, contributes to the pathogenesis of stroke development [5]. Studies showed that IR is associated with increased number of silent lacunar infarcts [5,13]. Besides the role in atherosclerosis development and progression, IR is associated with increased risk of venous thrombosis mainly driven by endothelial dysfunction and prothrombotic state [14]. The association between venous thromboembolism and atherosclerosis suggests common risk factors for both clinical conditions.

Patent foramen ovale (PFO), found in almost one fourth of the adult population, is an interatrial communication without any hemodynamic significance [15]. Over the past few decades, the association between PFO and ischemic stroke has become clear. Patients with cryptogenic stroke have been found to have a higher frequency of PFO than their healthy counterparts [16]. Although studies have emphasized paradoxical emboli through the PFO as a possible mechanism of ischemic stroke, most of the patients did not have deep venous thrombosis in their analyses [17]. In addition to paradoxical embolism, in situ thrombus formation and electrophysiological changes in atrial myocardium leading to atrial tachyarrhythmias or atrial fibrillation are the other proposed mechanisms that result in stroke in patients with PFO [18]. Anatomical characteristics of PFO, such as size, height, tunnel morphology, shunt degree, and presence of atrial septal aneurysm, have also been found to be associated with the occurrence of stroke in this group of patients [19].

The triglyceride glucose (TyG) index, calculated from plasma triglyceride (TG) and glucose levels, is an easily calculable and reliable index for assessing IR [20]. Unlike the homeostasis-model-assessment-estimated insulin resistance index, its calculation does not need to estimate plasma insulin levels. The predictive value of the TyG index has been shown in various diseases, including stable coronary artery disease, ischemic stroke, acute coronary syndrome, arterial stiffness, and heart failure [21,22,23,24,25]. Since IR is has been found as a risk factor for the development of stroke, we aimed to evaluate the value of the biomarker –TyG index—which is more easily calculated than the other parameters in PFO patients who experienced stroke. In this study, we sought to evaluate whether PFO patients who experienced embolic stroke have elevated TyG index levels compared to PFO patients without embolic stroke. In addition, we tried to evaluate the value of the TyG index in predicting stroke occurrence in patients with PFO.

## 2. Materials and Methods

### 2.1. Study Population

We retrospectively screened the files of patients who underwent transesophageal echocardiographic (TEE) examination between January 2020 and January 2022 in a tertiary hospital clinic. Patients with PFO who experienced embolic stroke constituted our study group. After selecting the patients in the study group, we chose subjects with PFO without stroke for the control group. A total of 108 and 129 patients were enrolled in the study and control groups, respectively. Patients with inflammatory diseases, coronary artery disease, diabetes mellitus, morbid obesity, malignancy, advanced hepatic/renal failure, lacunar brain infarct, triglyceride-lowering drug therapy, extra or intracranial atherosclerosis leading to more than 50% stenosis of arteries that were connected to the ischemic area, or an identified cause of stroke were excluded. All patients in the study group experienced embolic stroke within 2 weeks of enrollment. Ethical approval was obtained from the ethical committee of the tertiary hospital, and this study was conducted in accordance with the amendments of the Declaration of Helsinki.

All patients in the study group were referred from the neurology clinic for the clinical and anatomical evaluation of PFO. Patients were evaluated for other possible causes of stroke, and no identifiable cause of stroke was found.

### 2.2. Data Collection and Variables

The demographic and clinical features of the patients were obtained from the hospital data system. All the patients were evaluated for the possible etiologies of stroke, including lower-limb deep venous examinations, carotid Doppler ultrasonography, atrial fibrillation, and aortic atheromatous plaques. Carotid artery and lower-limb deep venous examinations were performed using Doppler ultrasonography. Diagnosis of PFO was made by TEE. Characteristics of PFO, including tunnel height and tunnel length, were assessed. In addition, a bubble contrast study using agitated saline was performed with Valsalva maneuver. Information regarding the investigation of possible etiologies of stroke were obtained from electronic healthcare records of the patients.

### 2.3. Definitions

Diagnosis of ischemic stroke was performed according to the World Health Organization guidelines (clinical status of the patients and the findings of computed tomography or magnetic resonance imaging methods) [26]. Stroke was defined as a clinical syndrome with rapidly evolving clinical symptoms or signs characterized by focal or global loss of brain function, with symptoms lasting more than twenty-four hours or earlier death, and without identifiable cause other than vascular origin. Ischemic stroke was defined as evidence of infarction on computed tomography or magnetic resonance imaging within the index event.

PFO was defined as evidence of the appearance of microbubbles in the left atrium within three cardiac cycles after their appearance in the right atrium during TEE. This appearance could occur at rest or during the Valsalva maneuver. The anatomic characteristics of the PFO were again assessed by TEE. A PFO tunnel length and height of more than 10 mm and 2 mm, respectively, and the presence of atrial septal aneurysm, a prominent Eustachian valve, or a Chiari’s network of more than 10 mm were considered high-risk PFO [27].

The Essen Stroke Risk Score was calculated for each patient. The ESRS was developed to predict recurrent stroke. The Essen Stroke Risk Score was obtained by summing the scores of risk factors, including age (2 points for age > 75 years, 1 point for age 65–75 years) and 1 point for hypertension, diabetes mellitus, previous myocardial infarction, cardiovascular disease, peripheral arterial disease, smoking history, and previous transient ischemic attack or ischemic stroke [28].

### 2.4. Blood Samples

Blood samples of the patients were collected after an overnight fast. Samples were centrifuged at 3000 rpm for 10 min. Complete blood count and biochemical assessments were carried out using automated blood cell counter (Coulter LH 780 Hematology Analyzer, Beckman Coulter, Inc., Galway, Ireland) and Siemens Healthcare Diagnostic Products kits and calibrators (Marburg, Germany).All samples were collected and analyzed within 2 weeks of stroke. Biochemical and complete blood count parameters were recorded. Serum glucose values were multiplied by TG values and then divided by two. The TyG index was calculated from a natural logarithmic transformation of the obtained result.

### 2.5. Statistical Analysis

The normality of the data was assessed by evaluation of skewness, kurtosis, and a Kolmogorov-Smirnov test. Comparisons of patients with or without stroke were performed using independent samples *t* tests or Mann-Whitney U tests, depending on the distribution of the data. Receiver operating characteristic (ROC) curve analysis was conducted to measure cutoff values of variables that predicted the occurrence of stroke. Comparison of the area under the curve (AUC) of the curves was performed using DeLong’s test. Quade ANCOVA was conducted to compare the TyG index between the two groups where age was used as a covariate. Univariate logistic regression was performed to identify the predictors of stroke. Variables that were found to be significant for the prediction of stroke were included in the multivariable logistic regression analysis. We conducted three types of multivariable logistic regression, model A, model B, and model C, with analyses of glucose, triglycerides, and the TyG index, respectively. A *p* value smaller than 0.05 was accepted as significant. All statistical analyses were performed. All statistical analyses were performed IBM SPSS version 23 software (the Statistical Package for the Social Sciences, Armonk, NY, USA). 

## 3. Results

The mean age of the study population was 44.95 ± 15.05 years, and 143 (60.3%) of them were male. Patients with stroke were significantly older (41 (30–54) years vs. 48 (42–55) years, *p* < 0.001), had higher values of glucose (95 (86–111) vs. 108 (95–128), *p* < 0.001), creatinine (0.72 (0.64–0.85) vs. 0.84 (0.74–0.96), *p* < 0.001), TG (114 (83–153) vs. 151 (106–187), *p* < 0.001), leukocytes (7.59 (6.21–8.91) vs. 7.88 (6.8–9.39), *p* = 0.025), TyG index (8.67 ± 0.49 vs. 9.91 ± 0.50, *p* < 0.001), Essen Stroke Risk Score, and lower high-density lipoprotein–cholesterol (HDL-C) (55 (46–59) vs. 44 (36.6–54.7), *p* < 0.001) values. A comparison of the two groups is shown in Table 1. According to the results of Quade nonparametric ANCOVA, the TyG index was significantly higher in patients with stroke when age was used as a covariate (t = −4.623, *p* < 0.001).

**Figure 1:**
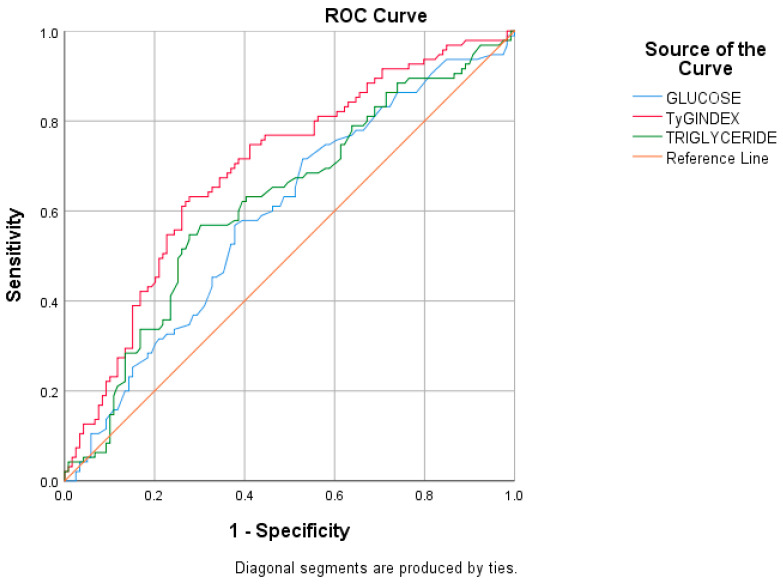
ROC curve analysis for the prediction of stroke.

The ROC curve analysis showed that the TyG index had the highest sensitivity for the prediction of stroke in comparison to TG and glucose values (63.2%, 54.7%, and 56.8%, respectively). The TyG index and TG had the highest specificity for the prediction of stroke (72.3%, for both) (Table 2, Figure 1). Comparison of ROC curves showed that the TyG index had the highest AUC compared to that of TG and glucose (0.694, 0.620, and 0.593, respectively) (Table 3). The TyG index value of 8.89 predicted stroke occurrence with a sensitivity and specificity of 63.2% and 72.3%, respectively.

Univariate logistic regression analysis showed that age (OR: 1.035), glucose (OR: 1.005), TG (OR: 1.019), creatinine (OR: 8.201), HDL-C (OR: 0.964), leukocyte count (OR: 1.135), and TyG index (OR: 3.913) were independent predictors for the occurrence of stroke (Table 4). We conducted three models of multivariable logistic regression analyses, in which triglycerides, glucose, and the TyG index were included in the analyses. The results of multivariable regression analyses showed that age, HDL-C, TG, and TyG index were independent predictors of stroke. The TyG index had a higher odds ratio than TG, which indicated that it had a better predictive value (2.827 vs. 1.006, respectively). Results of multivariate logistic regression analyses are shown in Table 5.

## 4. Discussion

Our results suggested that for the occurrence of stroke in patients with PFO, the TyG index, which has been considered a reflection of IR in the human body, had predictive value in these patients. Moreover, its predictive value was found to be higher compared to glucose and TG values, from which it was calculated.

Several studies have shown an association of causative factors between ischemic stroke and type II DM [29,30]. Most ischemic strokes are caused by embolic events. Compared with nondiabetic individuals, patients with type II DM had an almost fourfold higher risk of atherosclerotic vascular complications [31]. IR has been implicated as a key mechanism in the diagnosis of type II DM and, as such, has attracted the interest of researchers. Several epidemiologic studies found an association between stroke and IR, which is important because preventive measures such as lifestyle changes can help decrease the likelihood of this condition [32,33,34,35,36]. Various metabolic pathways have been implicated in the pathogenesis of stroke occurrence. IR has been found to be associated with decreased nitric oxide and increased endothelin-1 production, both of which lead to endothelial dysfunction, platelet activation, and thrombosis formation. The IR state is characterized by hypercoagulability and increased platelet number, volume, aggregation [37], and free fatty acid release, and this status is linked to an increased formation of coagulation factors and a prothrombotic state [8]. Ueno et al. found that IR and impaired glucose tolerance were the strongest predictors of platelet aggregation, which was proportional to the IR status [38]. The interaction between activated platelets and endothelium and leukocytes has been found to be facilitated in IR. This interaction is also important in the formation of atherosclerotic lesions and atherothrombotic complications [39]. By increasing inflammation and oxidative stress, IR could influence the modifiable risk factors for stroke [40,41]. As a surrogate marker for IR, the value of the TyG index in the prediction of both occurrence and adverse clinical outcomes for stroke has been shown in various clinical studies [25,42,43]. Yang et al. conducted a meta-analysis in order to find the association between TyG index and ischemic stroke in general population. They found that patients who had high TyG index values had increased risk of incident stroke, recurrent stroke, and death [42]. Similar to these findings, Feng et al. showed that risk of ischemic stroke was positively correlated with TyG index and independent risk factor for incident strokes [43].

It has been suggested that one of the mechanisms of PFO-related strokes is the passage of thrombi from venous circulation into systemic circulation. Any pathological deviation of the hemostatic system toward hypercoagulation might contribute to the formation of pathological thrombi [44]. Cerrato et al. described two patients with inherited coagulation disorders who experienced PFO-related embolic strokes during Valsalva-like maneuvers [45]. Chaturvedi showed that the prevalence of hemostatic abnormalities was comparably higher in patients with PFO-related strokes [46]. Pezzini et al. conducted a case-control study and searched whether an association exists between inherited thrombophilic disorders and PFO-related strokes [47]. In their study, they found that some thrombophilic mutations may be related to PFO-related infarcts. Lui et al. prospectively followed up 591 patients with PFO and cryptogenic embolism [48]. Patients with thrombophilia were found to be at higher risk for developing recurrent events. Plasma homocysteine levels, a risk factor for venous thrombosis, were found to be elevated in patients with PFO-related strokes [49,50].

To the best of our knowledge, our study was the first to evaluate the TyG index in PFO-related strokes. Our results suggested that age, HDL-C, and TyG index levels were the independent predictors of stroke. Since the patients who experienced stroke were significantly older in our study, we performed a Quade ANCOVA analysis to eliminate the effect of age on TyG index values. It has been shown that aging is associated with an increase in fasting insulin levels, insulin resistance, and central obesity [51]. The mechanism underlying age-related insulin resistance in these subjects involves interactions of many risk factors, such as increased adiposity, decreased physical activity, coexisting medical conditions, insulin secretory defects, use of medications, decreased secretion of insulin, and reduced hepatic sensitivity to its actions [52]. After the Quade ANCOVA analysis, the TyG index remained elevated in patients with stroke, indicating that the TyG index increased in these patients independent of age. Moreover, age was one of the independent predictors for stroke occurrence. Several studies have shown that the risk of stroke increases with age, with a doubling of the risk for each decade after the age of forty-five and more than half of all strokes occurring after the age of sixty-five [53]. Soto-Camara et al. investigated the effect of different modifiable risk factors in different age groups. They showed that patients younger than 75 years of age had a higher number of modifiable risk factors such as smoking, alcohol consumption, sedentary lifestyle, whereas in patients older than 75 years of age, the most prevalent risk factor was hypertension [54]. Low HDL-C is a known risk factor for cardiovascular disease. HDL-C has antithrombotic, antioxidant, anti-inflammatory effects and is the lipoprotein responsible for reverse cholesterol transport [55]. Low level range of HDL-C is associated with increased risk of stroke [55]. The relationship between HDL-C levels and stroke has been investigated in several studies, and the results have been controversial. Some of them found an inverse relationship between HDL-C levels and the occurrence of stroke [56,57]. Whereas others have shown that patients with extremely high levels of HDL-C have an increased risk of both ischemic and hemorrhagic stroke [58]. In our analysis, increasing levels of HDL-C was associated with a decreased stroke risk. Although the patients in the study group had higher LDL-C levels, it did not reach statistical significance. We found that in addition to other risk factors that have been investigated in previous studies, the TyG index was one of the predictors of the occurrence of stroke in PFO patients. The combination of both modifiable and non-modifiable factors could result in cardioembolic stroke in PFO patients [59]. A prothrombotic state affecting the coagulation cascade on the endocardial surface of the heart could be the starting event of thrombus formation [60]. As such, patients who had anatomical and additional predisposing conditions would have an increased risk of subsequent embolism [59,61]. According to our study, the TyG index might be a marker for the increased risk of stroke in PFO patients.

Limitations: Our study was a single-center and retrospective study. Long-term follow-up of the patients and the effect of TyG levels on the prognosis of the patients were not evaluated.

## 5. Conclusions

In patients with PFO, in addition to anatomical factors related to PFO, metabolic factors could affect the occurrence of stroke. The TyG index, an easily calculated and simple biomarker, was an independent factor for stroke in PFO patients. Assessment of the TyG index in cryptogenic stroke patients with PFO can help manage treatment options.

## Figures and Tables

**Table 1:** Comparison of two groups.

	Control Group (n = 119)	Study Group (n = 95)	*p*
Age (years)	41 (30–54)	48 (42–55)	<0.001
Gender (n, %)			0.207
Female	52 (43.7)	33 (34.7)	
Male	67 (56.3)	62 (65.3)	
Body mass index (kg/m^2^)	27.68 ± 4.32	27.01 ± 4.53	
Smoking (n, %)	72 (60.5)	55 (57.9)	0.780
Hypertension (n, %)	63 (52.9)	40 (42.1)	0.131
Hyperlipidemia (n,%)	57 (47.9)	40 (42.1)	0.411
Glucose (mg/dL)	95 (86–111)	105 (93–123)	0.020
Creatinine (mg/dL)	0.72 (0.64–0.85)	0.84 (0.74–0.96)	<0.001
LDL-C (mg/dL)	119.01 ± 35.82	121.17 ± 40.77	0.736
HDL-C (mg/dL)	55 (46–59)	44 (36.6–54.7)	<0.001
Triglyceride (mg/dL)	114 (83–153)	143 (99–148)	0.003
Hemoglobin (g/dL)	13.4 (12.6–14.6)	14.1 (12.9–15.1)	0.053
Leukocyte (10^3^/uL)	7.59 (6.21–8.91)	7.88 (6.8–9.39)	0.025
Lymphocyte (10^3^/uL)	2.23 (1.81–2.87)	2.32 (1.91–2.89)	0.288
Platelet (10^3^/uL)	247 (210–294)	253 (222–297)	0.261
TyG index	8.67 ± 0.49	9.01 ± 0.50	<0.001
Essen Stroke Risk Score	1.25 ± 0.88	2.14 ± 0.88	<0.001

HDL-C: High-density lipoprotein–cholesterol, LDL-C: Low-density lipoprotein–cholesterol, TyG index: Triglyceride–glucose index.

**Table 2:** ROC curve analysis for prediction of stroke.

	AUC	*p*	95% CI	Cut-Off	Sensitivity	Specificity
Triglyceride	0.620	0.003	0.544–0.696	138.5	54.7	72.3
Glucose	0.593	0.020	0.516–0.669	102.5	56.8	62.2
TyG index	0.694	<0.001	0.623–0.765	8.89	63.2	72.3

TyG index: Triglyceride–glucose index.

**Table 3:** Comparison of ROC curves of triglyceride, glucose, and TyG index.

	z	*p*	AUC Difference	95% CI
Triglyceride-Glucose	0.494	0.622	0.027	−0.81–0.135
Triglyceride-TyG index	−2.582	0.010	−0.074	−0.130–−0.018
Glucose-TyG index	−2.357	0.018	−0.101	−0.185–−0.017

TyG index: Triglyceride–glucose index.

**Table 4:** Univariate logistic regression for prediction of stroke.

	*p*	OR	95% CI
Age	0.001	1.035	1.015–1.056
Glucose	0.295	1.005	1.001–1.021
Triglyceride	0.019	1.019	1.003–1.012
Creatinine	0.004	8.201	1.948–34.525
HDL-C	0.001	0.964	0.944–0.985
Leukocyte	0.047	1.135	1.001–1.287
TyG index	<0.001	3.913	2.154–7.110

HDL-C: High-density lipoprotein–cholesterol, TyG index: Triglyceride–glucose index.

**Table 5:** Multivariate logistic regression analyses for prediction of stroke. Model A included glucose, Model B included triglyceride, Model C included TyG index.

Model A			
	*p*	OR	95% CI
Age	0.030	1.025	1.002–1.047
Creatinine	0.064	4.284	0.917–20.005
HDL-C	0.006	0.971	0.950–0.992
Leukocyte	0.207	1.090	0.953–1.248
Glucose	0.802	1.004	0.989–1.008
Model B			
	*p*	OR	95% CI
Age	0.026	1.025	1.003–1.048
Creatinine	0.083	3.968	0.834–18.874
HDL-C	0.013	0.973	0.952–0.94
Leukocyte	0.228	1.087	0.949–1.245
Triglyceride	0.045	1.005	1.001–1.009
Model C			
	*p*	OR	95% CI
Age	0.049	1.023	1.000–1.046
Creatinine	0.139	3.242	0.682–15.406
HDL-C	0.049	0.978	0.957–1.000
Leukocyte	0.330	1.071	0.933–1.228
TyG index	0.001	2.872	1.524–5.412

HDL-C: High-density lipoprotein–cholesterol, TyG index: Triglyceride–glucose index.

## Data Availability

The data that support the findings of this study are available on request from the corresponding author.
